# Frequency and Phase Characteristics of Candle Flame Oscillation

**DOI:** 10.1038/s41598-018-36754-w

**Published:** 2019-01-23

**Authors:** Ting Chen, Xiao Guo, Ji Jia, Jinghua Xiao

**Affiliations:** 1 School of Science, Beijing University of Posts and Telecommunications, Beijing, 100876 China; 2 State Key Lab of Information Photonics and Optical Communications, Beijing University of Posts and Telecommunications, Beijing, 100876 China

## Abstract

The combustion of candles exhibits a variety of dynamical behaviors. Binding several candles together will result in flickering of candle flames, which is generally described as a nonlinear oscillator. The impact on the frequency of the flame by several factors, such as the arrangement, the number and the asymmetry of the oscillators, is discussed. Experimental results show that the frequency gradually decreases as the number of candles increases in the case of an isolated oscillator, while alternation between the in-phase and the anti-phase synchronization appears in a coupled system of two oscillators. Moreover, envelopes in the amplitude of the oscillatory luminance are displayed when candles are coupled asymmetrically. Since the coupling between oscillators is dominated by thermal radiation, a “overlapped peaks model” is proposed to phenomenologically explain the relationship between temperature distribution, coupling strength and the collective behavior in coupled system of candle oscillators in both symmetric and asymmetric cases.

## Introduction

The proficiency of utilizing fire made it possible for homo-sapiens to get rid of the dark and cold, moving out of caves and becoming the most developed species in the world. Candles, derived from ancient torch, have a prolonged history of usage for the purpose of illumination dating back to early civilization. The great availability, inexpensiveness and stability make candles ideal for people to explore features of diffusion flames. With the help of high speed camera, the complex dynamics underlying candle flames could be recorded and measured nowadays. In previous works, the candle flames were found to be able to spontaneously crowd together and exhibit limit-cycle oscillation^1–3^. In natural and engineering science, similar systems of limit-cycle oscillators were observed and discussed comprehensively, such as the synchronization in the flickering of fireflies^4,5^, rhythms in applause of crowd^6^, trends in stock markets^7^, swing of the pendulum^8^, oscillation of inverted bottle oscillators^9^ and so on. Abundant collective behaviors have been observed in systems of coupled oscillators, including various synchronizations^10–14^, amplitude death^15–21^ and the formation of spatial-temporal patterns^22–24^. Studying on the coupled oscillatory systems will be useful to the understanding of nonlinear dynamical behavior such as synchronization and emergence. Furthermore, the control of fire is worthy of investigation in order to avoid the deflagration and instability of combustion and flame.

The nonlinear oscillation of candle flames was introduced and analyzed with imaging technique by Chamberlin *et al*. in 1948 for the first time^25^. Decades later, two groups of burning candles were investigated with video clips by Kitahata *et al*. where the oscillation mainly consists of two modes depending on the distance between these oscillators^1^. The in-phase synchronization was observed when two groups were closely placed, while a distance further enough led the system to the anti-phase synchronization. According to the previous researches, the thermal radiation was considered as the main cause of the coupling between flames, and a theoretical model was proposed, which emphasizes the importance of distance and typical modes of flame oscillation. Since then, various experiments on coupled flaming candles have been designed. A number of oscillation modes with different spatial separations and arrangement topology were observed by Forrester in 2015^2^. Following the initial work of Forrester where three candles in an equilateral triangular arrangement, amongst others, was examined. Okamoto *et al*.^3^ investigated three candle groups with equilateral triangular arrangement in detail, and discovered four distinct oscillation modes: in-phase synchronization, partial in-phase synchronization, rotation and death. The frequencies of occurrence of these modes with disparate side lengths were computed and explained by vortex and bifurcation theories.

In this work, three key features of the flame oscillation were investigated. Firstly, a negative linear correlation between the number of the candles tied up in a single oscillator and its frequency is discovered. Furthermore, the impact of different arrangements of candles is studied on the amplitude and the frequency. Secondly, we analyzed the coupled system of two identical oscillators with an infrared camera to measure the temperature distribution in a flame and especially focus on the in-phase and anti-phase oscillation. A concise and vivid “overlapped peaks model” is proposed to explain the coupling interactions between flames with a phenomenological perspective. As will be seen, the width of the temperature distribution curve of a single oscillator reflects its effective radiation range, while the overlapping region of two coupled oscillators reflects the coupling strength. In-phase mode appears only when the oscillators are close enough to maintain coupling with each other all the time. Other modes appear when coupling strength remains stable for a minimum amount of time, resulting in phase-locked synchronization. The flames oscillates asynchronously when they are far enough apart, as the coupling strength diminishes. Finally, the model is extended into a system with two non-identical oscillators, where the asymmetric structures are found to cause imperfect in-phase and anti-phase oscillations. The weaker oscillator will be subordinate to the stronger one and supply a smaller radiation range, which leads to a deviation from the pure in-phase or anti-phase synchronization. When the distance is large enough, the phase difference will drift continuously due to the lack of coupling. The model proposed aims to explain how the distance between candle oscillators leads to multiple different collective behaviors of them.

## Methods

All candles in our experiments are made of paraffin, with a diameter of 6 mm and height of 60 mm. The initial size of each candle is considered the same. If not stated otherwise, candles in a group are tightly bound with tapes into a compact structure, and a flame oscillator contains three candles. Our experiments are performed in open air at indoor temperature and without ambient light and external air flow.

The image sequences of our experiments were recorded by a high speed camera (Cube4, Mikrotron, Germany). A 10-second gray-scale movie was recorded at 480 frames per second and hence converted into 4800-frame image sequence with MATLAB to investigate the oscillation of flames’ luminance. The amplitude is calculated by integrating the brightness (gray-scale values) in an adequate area for each frame. The brightness (at any moment in time) in those figures are indicated by the mean gray-scale value of each frame with fixed camera parameters. Thus, the brightness is dimensionless. Temperature curves are obtained with an infrared camera (FLIR ONE, FLIR, U.S.A.) against a black background with diffusive reflection. The obtained infrared images are converted into temperature matrices with FLIR TOOLS, which are plotted as curves after computing the mean value of each column of the matrix. Schlieren images are obtained with a single-mirror Schlieren system, in which the concave spherical mirror has a focal length of 1000 mm and diameter of 203 mm.

## Results

### The influence of the number of candles on a single oscillator

**Figure 1:**
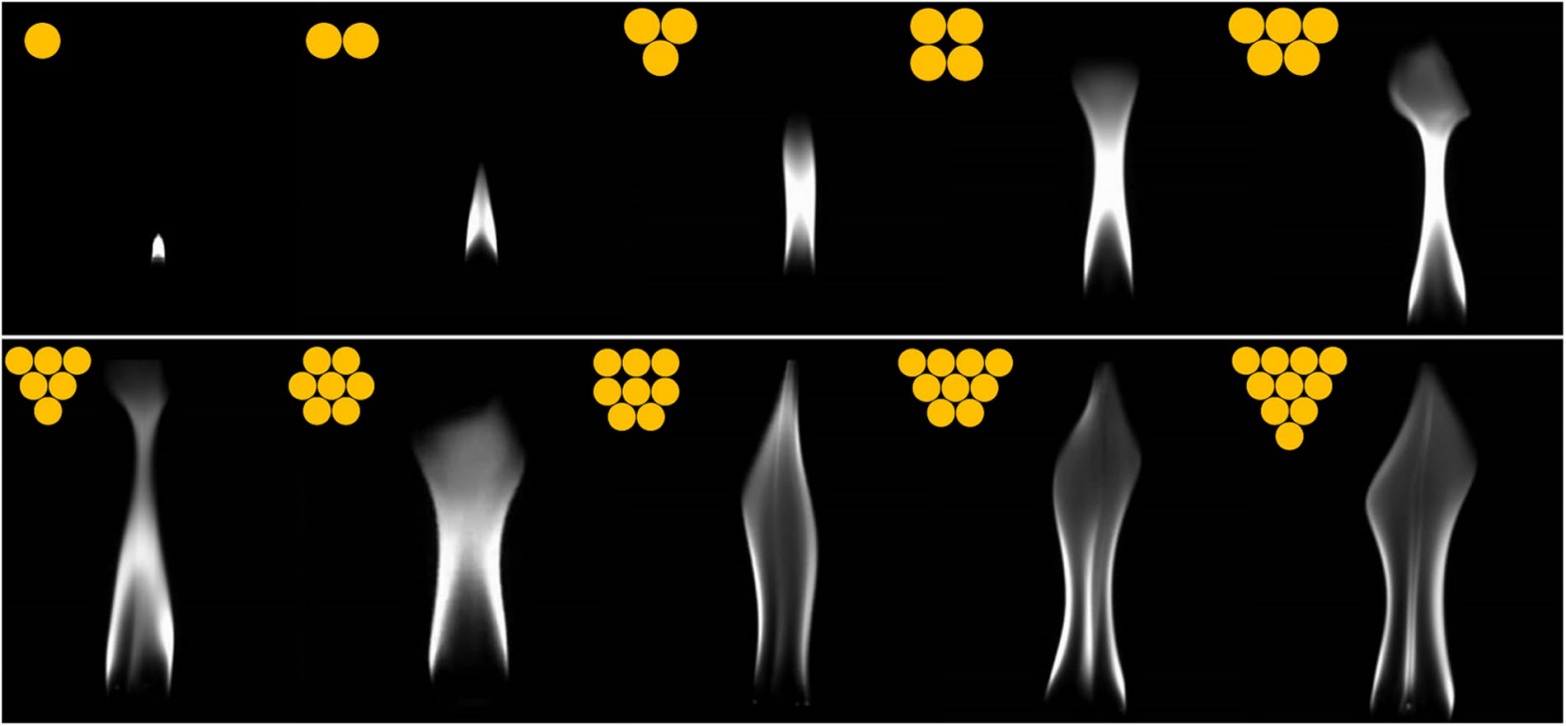
Gray-scale images of 1 to 10 candles. As the number increases, the flame becomes larger in width and height. The dots on the upper left represent the arrangement of bound candles in each group.

**Figure 2:**
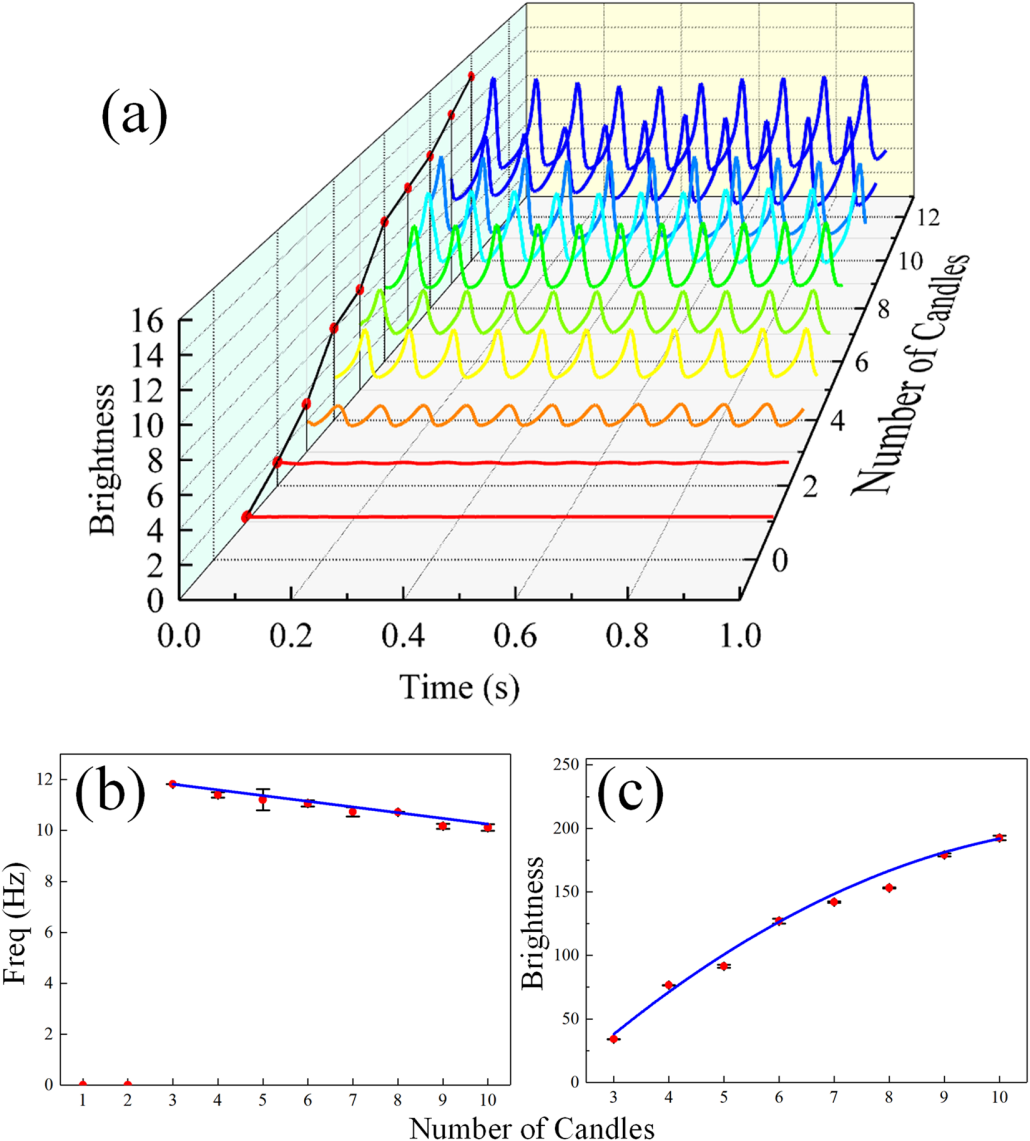
(**a**) Time series of the flame brightness with different number of candles in a group. As the number increases, the amplitude and average value of brightness increases dramatically. (**b**) Number — Frequency diagram. When the number is less than 3, frequencies are null; when the number is 3 or more, frequencies decrease monotonically. The blue line was a linear fit. (**c**) Number — Brightness diagram. The brightness is the average value in a single period for each group. The brightness increases as the number increases. Both of the error bars stand for standard deviation of six repetitive experiments.

Kitahata *et al*. pointed out that the flame of a single candle oscillator would flicker periodically when it consists of no less than 3 candles. Otherwise, it maintains stable combustion. Thus, the origin of the oscillation and the impact of the number of candles in an oscillator deserve detailed investigation. Flame oscillators containing from 1 to 10 candles were experimentally tested. The arrangement of candles is indicated by the yellow dots in Fig. 1. The high speed camera is aligned with the center of candle flames with the distance between them fixed. All the footages are recorded when the flame reaches a stable oscillation state and, as shown in Fig. 1, the gray-scale images display the peaking moment of each group of flame. The flame profile varies in amplitude, which generally tends to increase monotonically with the number of candles. For a single candle, the flame does not exhibit visible oscillation and remains stable; for a group of 2 candles, the brightness of the flame increases slightly and the flame exhibits tiny fluctuation at times, yet neither regularly nor obviously. For the group consisting of more than 3 candles, the flame exhibits regular oscillation which has more or less stable amplitude and frequency. As the number of contained candles increases, the brightness increases monotonically as well. Time series are obtained (see in Methods section) and shown in Fig. 2(a). The frequency spectrum of each oscillator is obtained by Fast Fourier Transformation (FFT) and its dependence on the number of candles is shown in Fig. 2(b). When the number is less than 3, the flames remain stable yet non-periodic. When the number is equal to or greater than 3, oscillation appears and the frequency monotonically decreases as the number increases. Moreover, the frequency remains in the range of 10–12 Hz, which expectedly matches the results of T. Maxworthy and Hamins *et al*.^26,27^, in which diffusion flames were concerned and the frequency was determined by the diameter of jets and the strength of the flow. The data fit an empirical formula between the frequency and the burner diameter^28^: f ∝ D^−0.49^.

When the number of candles contained increases, the fuel flow rate accordingly increases and thus leads to the growing demand for oxygen. The open air around the burning candles has a rather low flow rate^29^, which can be viewed as quasi-static. It takes more time to replenish the needed air to the burning region when the reaction is more drastic. Meanwhile, the puff generated by the candles becomes larger as the number increases, requiring a longer time to float upward into the open air. In consequence, the frequency of oscillator decreases with the increasing number.

**Figure 3:**
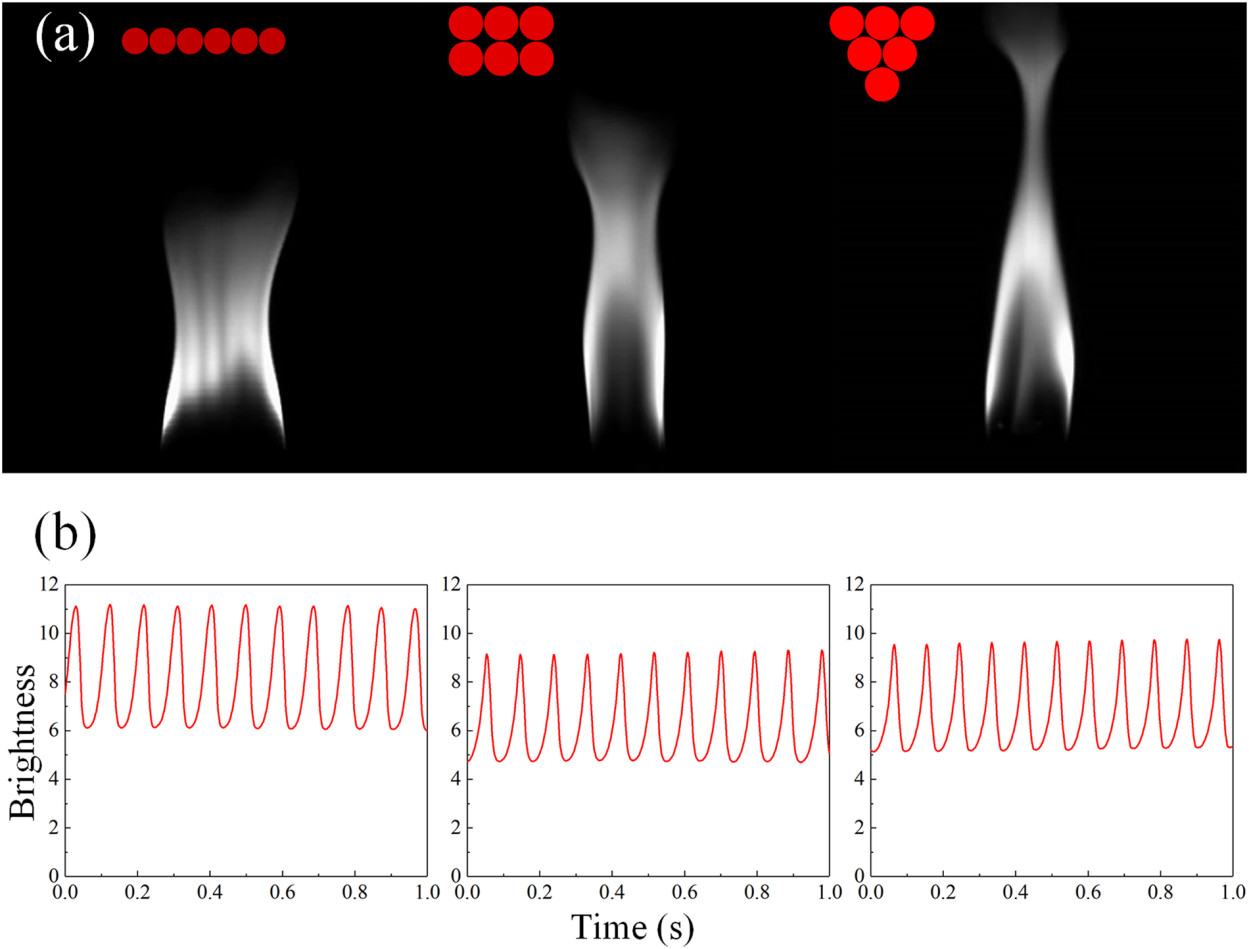
Different arrangement of 6 candles in a group. (**a**) Gray-scale images and (**b**) time series of each type. Corresponding frequencies are 10.7227 Hz/10.7802 Hz/10.9570 Hz (left to right).

It is noteworthy that arrangement affects the oscillation behavior as well, even with the same number of candles in an oscillator. In the case of 6 candles, for instance, three types of arrangement are checked in our experiment, and it is found that the brightness and the frequencies are all different. The first type, as shown in the left of Fig. 3(a), has the largest amplitude and smallest frequency due to its greatest width. On the other hand, the most closely arranged group has the highest frequency but smallest amplitude, since a smaller reaction surface will result in both less consumption of oxygen and smaller puff as mentioned above. However, the difference in these three cases are not significant in reality, which indicates that the impact of arrangement is much weaker than the number of candles.

### Synchronization between two identical symmetric oscillators

**Figure 4:**
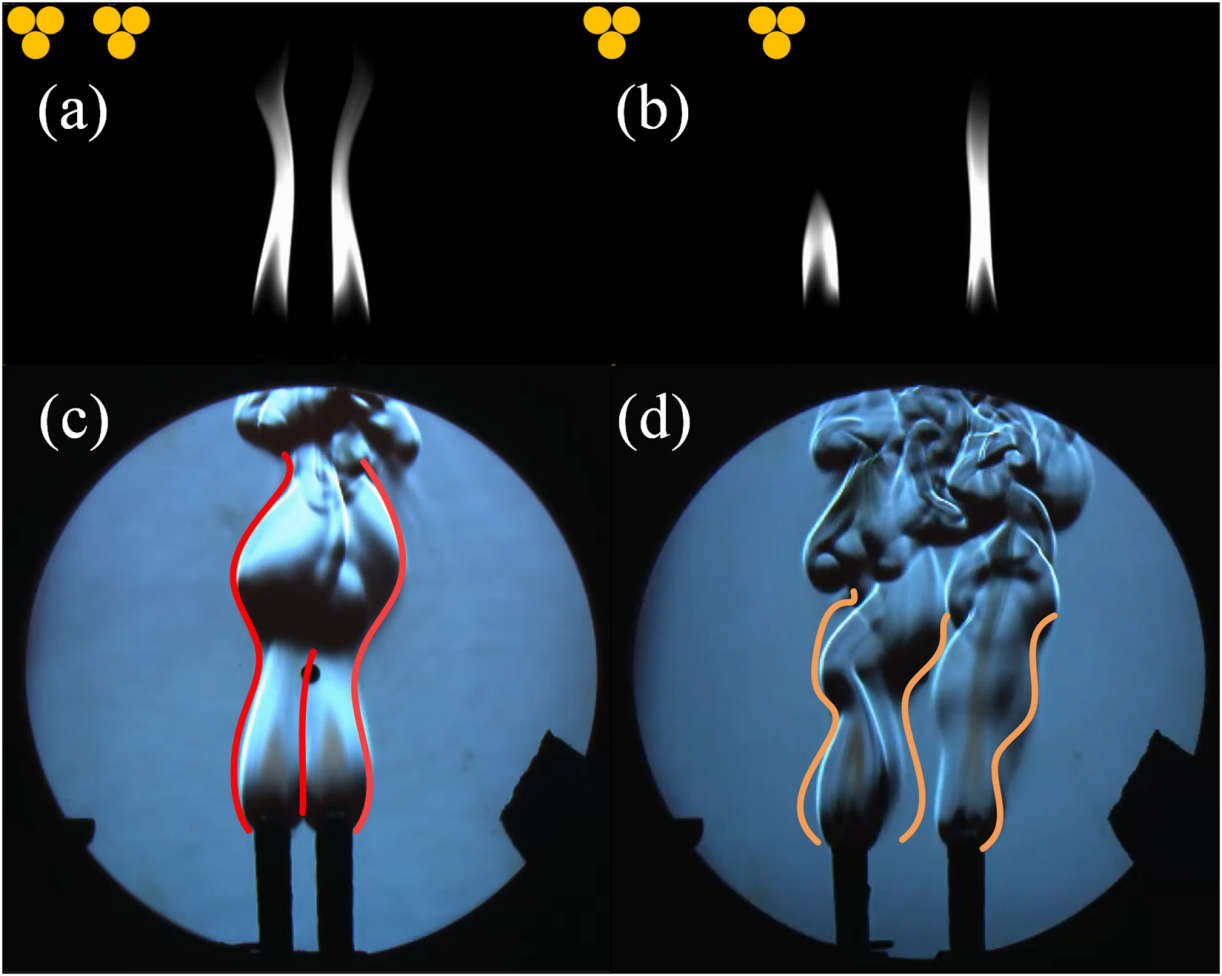
(**a**) The gray-scale image of the in-phase (20 mm between two oscillators, left) and (**b**) anti-phase synchronization (35 mm, right).(see Supplementary Vids S1 and S2) (**c**) Schlieren images of the in-phase mode (see Supplementary Vid. S3), and (**d**) anti-phase mode.

The impact of the number of the candles and the arrangement on oscillation amplitude and frequency for a single oscillator is discussed in the previous section. In this section, we investigate a coupled system of two identical oscillators. Kitahata *et al*. found that two flame oscillators exhibited the in-phase synchronization when the distance between them is between 20 mm and 30 mm and the anti-phase synchronization for the distance between 30 mm and 48 mm. In our experiments, the distance between the candles is set to 20 mm initially but ends at 60 mm, with a step size of 5 mm. Figure 4 shows the gray-scale images of the in-phase and anti-phase oscillation. As the distance increases, the synchronization state of the system changes from the in-phase to the anti-phase at about 35 mm and from the anti-phase to the incoherent at 60 mm. The relationship between the distance and the frequency of the oscillators is recorded and analyzed, and comply well with former result^1^. The frequency increases slightly when the system is in-phase synchronized, but decreases from a high frequency in the anti-phase. Furthermore, Schlieren images were presented in order to investigate the synchronization states between candle groups. Comparing the flow patterns of in-phase and anti-phase synchronization, we can make a distinction between them. As for the in-phase mode, the outline of the flow pattern shows spatial symmetry and the inner profile is close to a straight line. Asymmetrical curves are observed for the outline and the inner line when it comes to anti-phase mode. The observation of flow patterns can provide another perspective of distinguishing the synchronization modes.

**Figure 5:**
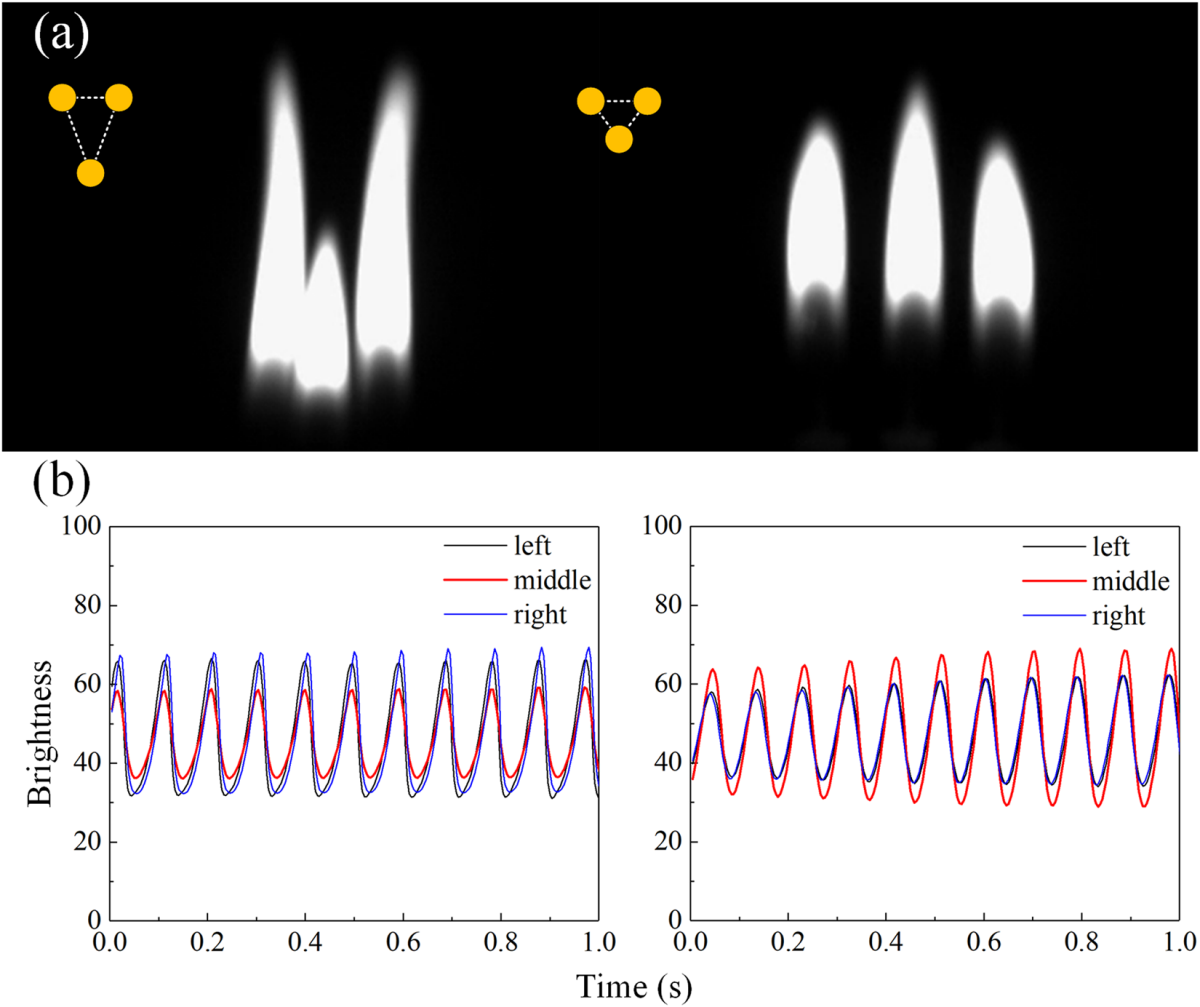
(**a**) Gray-scale images of three candles arranged in isosceles triangle with bases of 2 cm. The dots on the upper left represent the arrangements. The apex angle of the left is 39 degrees (<60 degrees), and the right one is 120 degrees (>60 degrees). (**b**) Time series of the brightness. That of the apex candle (sitting in the middle in the gray-scale images) is plotted with red curves which have lowest or highest amplitudes respectively, and the others are black and blue.

After the study of the symmetrically coupled system of two oscillators, we proceed to the system of three candles positioned in isosceles triangle. When the distances between them are small enough, each single candle in the triangle which burned stably starts to oscillate and shows in-phase synchronization with each other. As shown in Fig. 5, a smaller amplitude of the flame oscillation is observed on the candle sitting at the apex when this angle is smaller than 60 degrees, and a larger amplitude is seen for an apex angle greater than 60 degrees. According to our analysis, the difference is associated with different coupling strengths. The coupling strength consists of heat radiation and heat flux^1^, as well as vortex driven airflow^3,29^. Closer distance leads to higher temperature between flames and higher velocity of the vortex, which lead to greater impact on coupling strength. In the former case, the triangle has two long sides and a short base. Therefore, the candle at the apex is weakly coupled to the other two and has lower amplitude, while in the latter case the coupling becomes relatively stronger which leads to higher amplitude.

In our experiments, we focus on the impact generated by heat radiation, which is positively correlated with temperature. Hence the measurement of the temperature between flames can indicate the coupling strength between oscillators. Since the radiation flux decays with an inverse square law in distance, we suppose that for a single oscillator, there is an effective radiation range in which another flame is considerably influenced while the effect of radiation can be ignored outside. The higher temperature, the greater coupling strength and vice versa. When it drops down to near ambient temperature, the oscillators cannot maintain their coupling. Therefore, the coupling strength decreases monotonically with the increasing distance between candles, which will be used to forge a phenomenological explanation of the results later.

**Figure 6:**
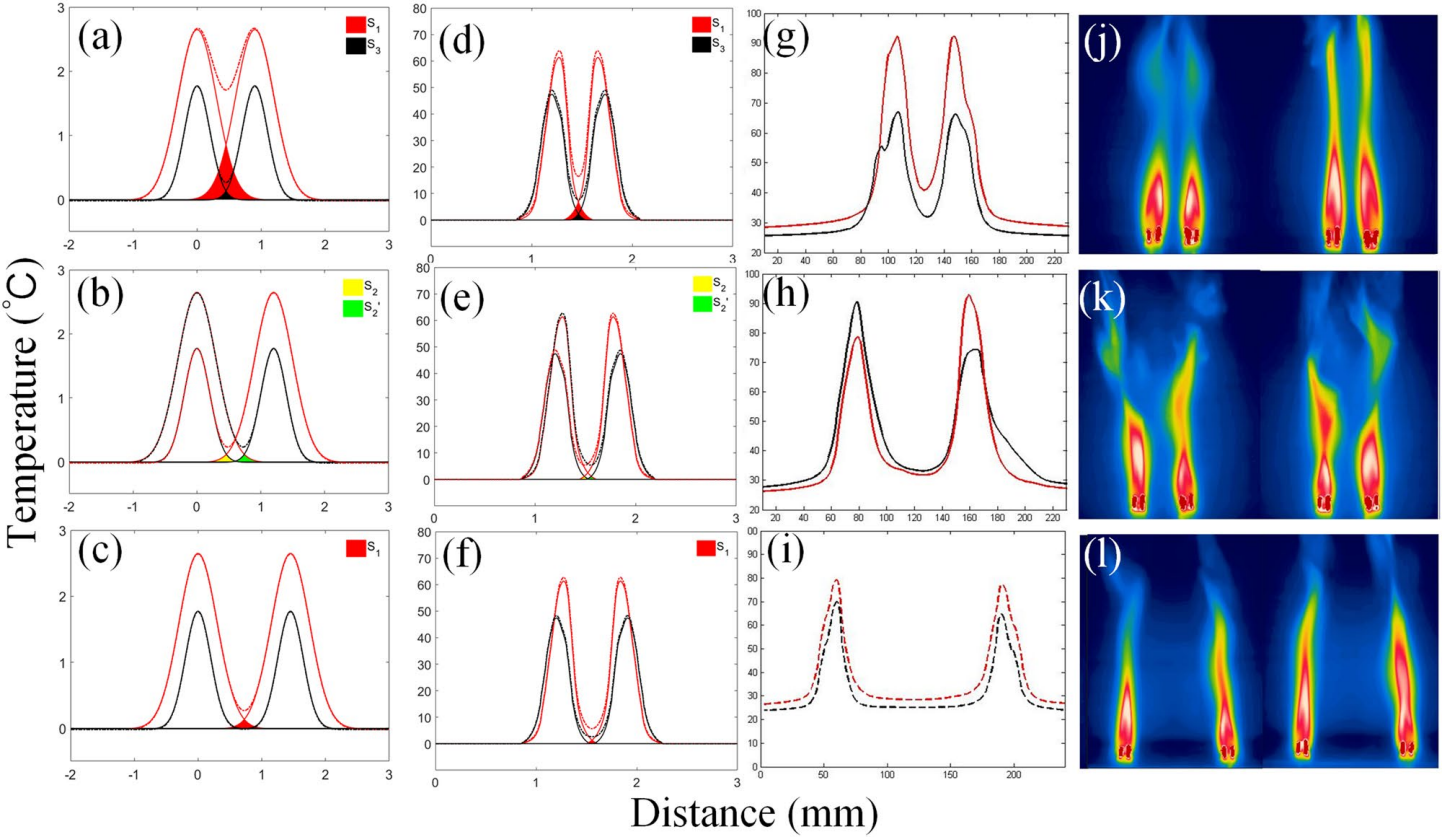
Phenomenological explanation of the mechanism of synchronization in the symmetric system. Each column is arranged in the order of in-phase, anti-phase and incoherent solution, as the distance increases. (**a**–**c**) Phenomenological model curves. (**d**–**f**) Simulation curves using the data of temperature distribution of single group containing 3 candles. (**g**–**i**) Real temperature distribution curves. (**j**–**l**) Infrared images.

Many researches have shown that when the coupling strength gradually changes between coupled oscillators, there exists a threshold value^30–34^ for the transition of synchronization states, or the basin stability of coherent states changes along with the change of coupling strength^35^. Considering the experiments of two identical oscillators, we might intuitively arrive at a conclusion that the coupling strength should decay along with the augmentation of distance between them. When decayed to a certain point, the synchronization state should switch from coherent to incoherent. However, this intuition does not comply with the result shown in Fig. 6. When the distance increases, the state turns from in-phase to anti-phase synchronization. This means that the transition of states is not caused by the change of basin. Therefore, the cause of the state transition deserves further research.

Considering the coupling led by the thermal radiation between flame oscillators, the temperature distribution between two oscillators was probed with the help of infrared camera. Figure 6(j–l) depict the case of in-phase (20 mm between two oscillators), anti-phase (40 mm) and incoherent (70 mm) oscillation. Based on all these experimental observations, “overlapped peaks model” was proposed to explain the phenomena. With the help of the model, we could link the change of distance with the transition of synchronization states. The model was shown in Fig. 6 and described as follows. As shown in Fig. 6(a–c), the red solid line represents the range at the maximum radiation and the black stands for the range at the minimum. Both of the lines are Gaussian curves. The horizontal axis indicates a negligible radiation strength. For the coupled oscillators, the strength of coupling is represented by the overlapping area below the two effective radiation curves. The maximum and minimum radiation curves are the key point of the model. Obviously, in case of two coupled flames, there will be four overlapping domains constituted by these two pair of curves. The overlapping domain of the two minimum profile is filled with black and marked as S3, and the maximum overlapping marked with red and S1, as shown in Fig. 6(a); the yellow (green) domain, marked as S2(S2′), indicated the overlaps constituted by one flame reaching its maximum (minimum) curve and the other one getting its minimum (maximum) curve, as shown in Fig. 6(b) for instance. It should be noted that these domains may be covered by each other. Thus, to ensure the definition of each domain, not all of them are shown in each sub-figure. For instance, in Fig. 6(a), the S1 domain is partly covered by S3, and S2 and S2′ are not expressed while they are exist indeed. When the oscillators are close enough, the relationship of S1 > S2 > S3 > 0 is satisfied as shown in Fig. 6(a). That is to say, even if the two flames fall to their minima, the system still has an adequate coupling to maintain the in-phase synchronization. As the distance increases, S3 domain vanishes, hence S1 > S2 > 0 = S3 as depicted in Fig. 6(b). In this case, flames cannot keep strong enough coupling to maintain the coherence if both reach the minimum while in the anti-synchronization the two flames alternatively reach the minimum and are able to maintain the coupling and the coherence. When the distance is small enough, S1 > 0 = S2 = S3 as shown in Fig. 6(c). In this situation, flames can keep neither in-phase nor anti-phase synchronization, since the coupling strength is not strong enough for most of the time, and the oscillation becomes incoherent, i.e., the phase difference between two oscillators cannot be locked.

If the model proposed is correct, then the temperature curve and phenomena should accord with the prediction of the model. In order to verify our model, we took infrared images of a single group of candle flame when it reaches its maximum and minimum separately. The temperature distribution curve is then calculated and is considered as the effective radiation scope of a single oscillator. The ambient temperature is deemed to be the bottom asymptotic line for the curves, as the coupling strength on both sides is nullified when the curves decay to ambient temperature. We apply two sets of same curves to simulate the temperature distribution of the coupled system of two identical oscillators. Comparing these simulated curves (d–f) with the ones given by the model on the left (a–c) and real temperature distributions on the right (g–i), we have obtained consistent results via same plotting methods. These results indicate that our model provides a valid and meaningful prediction of the phenomena observed in the experiments. So far, based on this model, the synchronization state could be phenomenologically explained: when the oscillators are close enough to each other, positive feedback of heat radiation leads to in-phase mode; when distance becomes larger, the system need to keep a *π*-phase difference to remain its stability; when distance is large enough, the coupling strength is so weak that oscillators cannot cohere with each other no matter what phase difference.

### Synchronization between non-identical asymmetric oscillators and their phase difference

**Figure 7:**
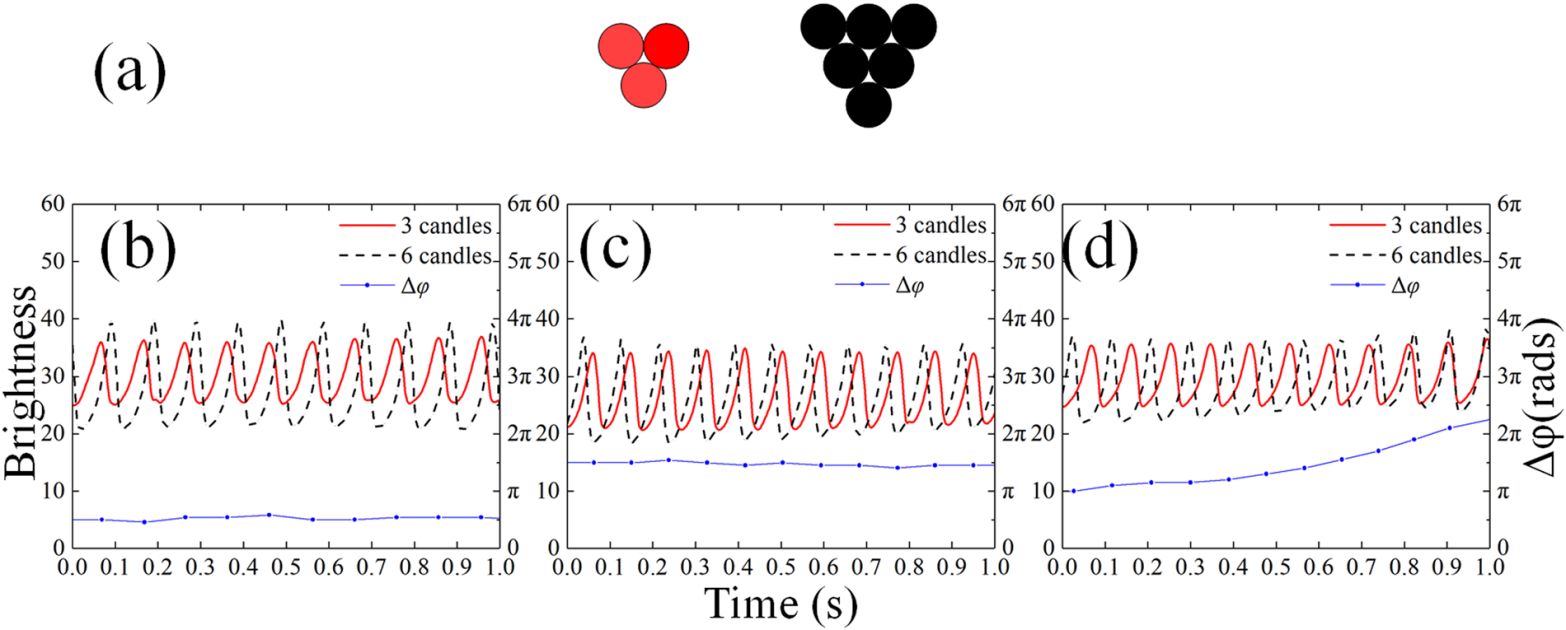
(**a**) Asymmetric arrangement of the “3 + 6” system. (**b**–**d**) Time series and phase differences. Black dashed lines for the 6-candle group, red solid lines for the 3-candle group and Blue dotted lines for the phase difference.(see Supplementary Vid. S4) (**b**) the near-in-phase synchronization (15 mm–35 mm), (**c**) the near-anti-phase synchronization(35 mm–55 mm), (**d**) the incoherent oscillation (>55 mm).

**Figure 8:**
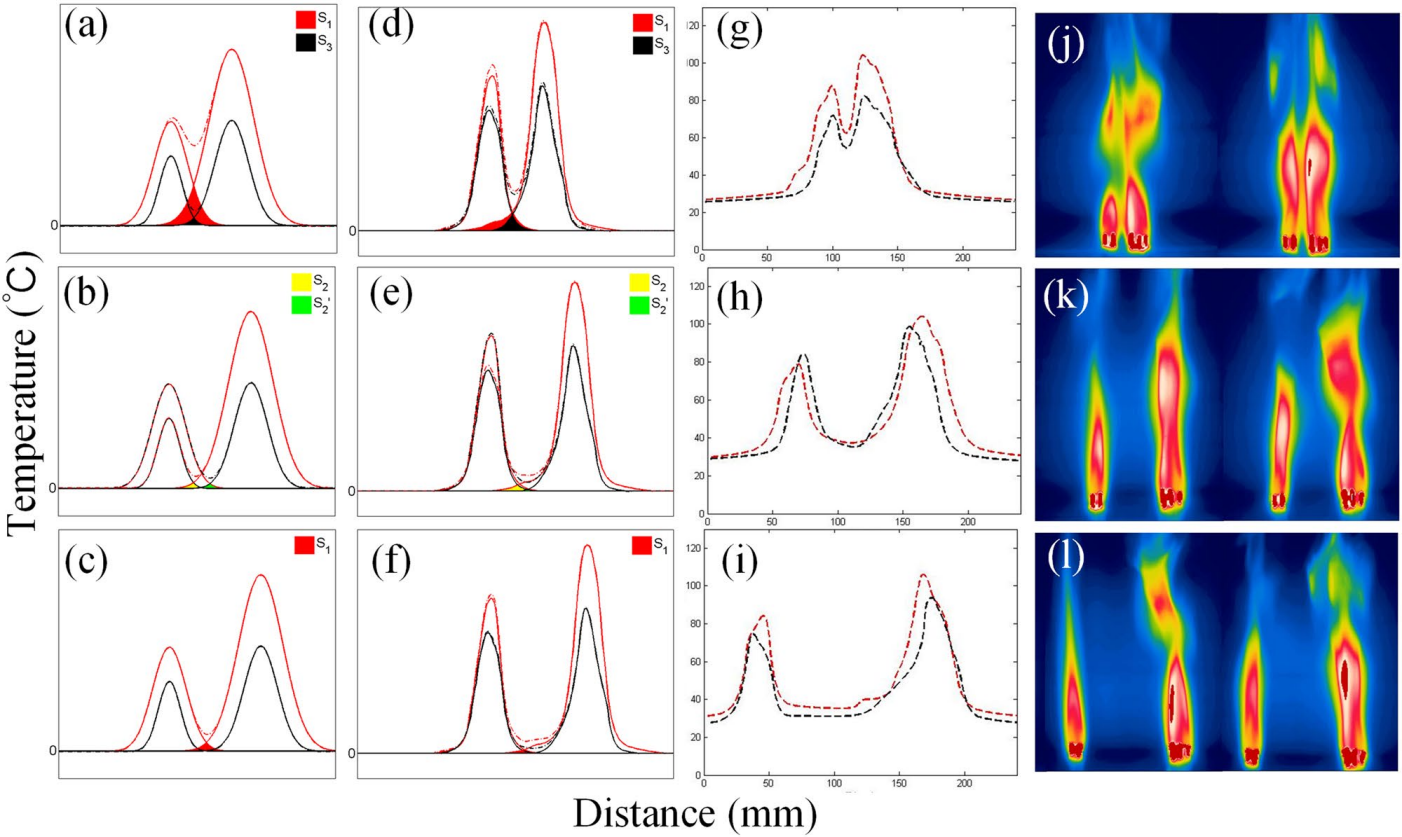
Phenomenological explanation on the mechanism of synchronization in the asymmetric system. Each column is arranged as the distance increases. (**a**–**c**) Phenomenological model curves. (**d**–**f**) Simulation curves using the data of temperature distribution of a single group containing 3 candles. (**g**–**i**) Real temperature distribution curves. (**j**–**l**) Infrared images.

**Figure 9:**
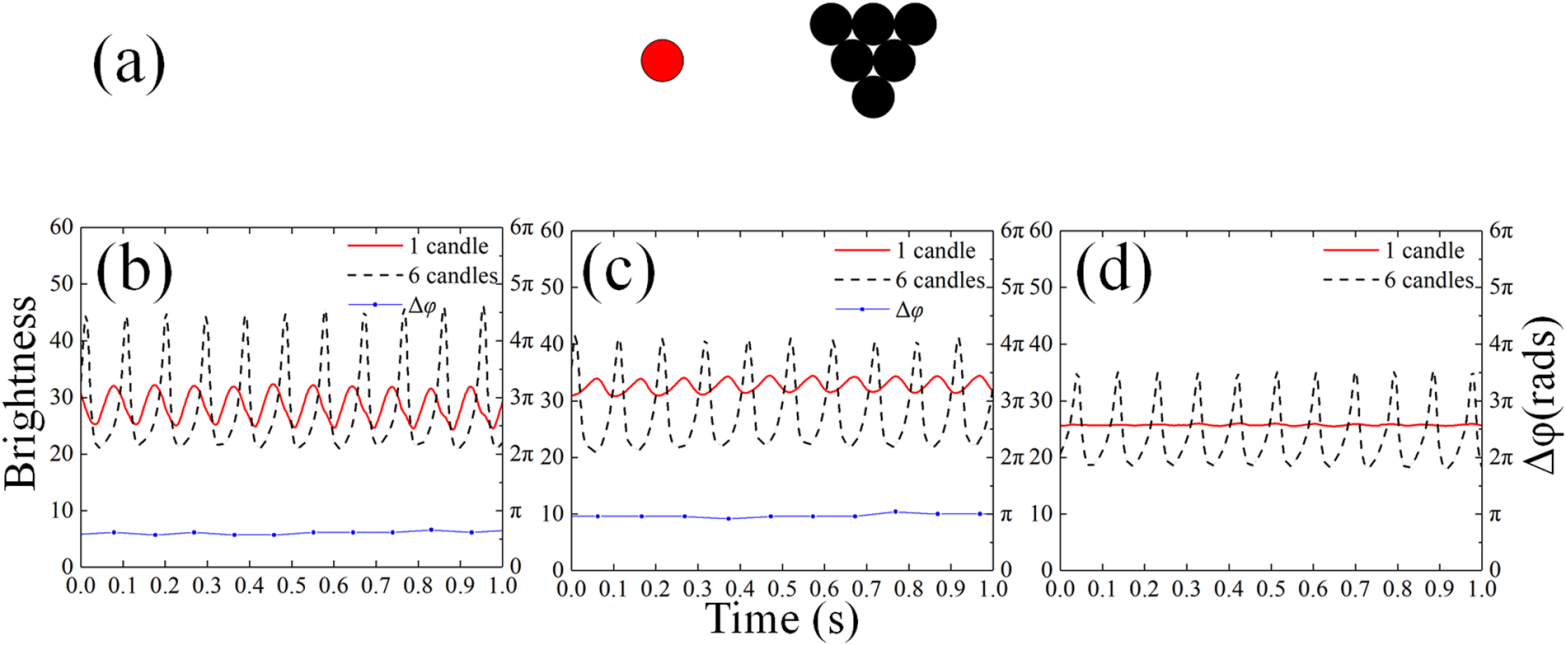
(**a**) Asymmetric arrangement of the “1 + 6” system. (**b**–**d**) The time series and phase differences. Black dashed lines for the 6 candles, red solid lines for the single candle and Blue dotted-lines for the phase difference. (**b**) Synchronization close to the in-phase (15 mm–35 mm), (**c**) synchronization close to the anti-phase (35 mm–55 mm), (**d**) the incoherent oscillation (>55 mm).

Several interesting phenomena are observed in symmetric coupled system, and in this section we study the coupled system of two non-identical oscillators. Two asymmetric systems are discussed. (1) The “3 + 6” pattern, which consists of an oscillator containing 3 candles and one containing 6 candles, as shown in Fig. 7(a), while the corresponding analysis is depicted in Fig. 8. (2) The “1 + 6” pattern, which consists of an oscillator with one single candle and another with 6 candles, as depicted in Fig. 9(a).

We start with the “3 + 6” pattern. Similar to the symmetric system, the flames were synchronized and phase-locked. When the flames are very close (15 mm–35 mm in our experiments), however, the phase difference is no longer zero due to its asymmetry. As the distance increases (35 mm–55 mm), the system turns to the phase locked synchronization close to the anti-phase. When the distance is larger than 55 mm, the flames become incoherent and the phase difference changed continuously. Figure 7(b–d) show the time series for these cases. Same results are obtained when it comes to the frequency domain. The synchronization state close to the anti-phase has a higher frequency which decreases as the separation between oscillators grows, while the state close to the in-phase has lower but increasing frequency.

The “overlapped peaks model” can also be applied to the explanation of synchronization in an asymmetric system. Similar methods are been implemented, though some details are been altered. According to our model, the synchronization state should resemble in-phase mode when distance is smaller and anti-phase mode when larger. Also, the oscillation should be dominated by the larger “6” group who is stronger in coupling strength. In Fig. 8, the left scopes represent the emaciated oscillator containing 3 candles, while the right curves stand for the sturdy one possessing 6 candles accordingly. In contrast to the symmetric cases, the effective radiation scopes of “3” and “6” are not identical, hence the overlapping domains are also not symmetric neither, especially for the areas of S2 and S2′ which determine the coupling strength to the other and are not equal any more. For the case that S1 > S2 (>S2′) > S3 > 0, the oscillator of “6” will impose stronger coupling strength on “3” apparently (which means “6” has higher temperature or stronger radiation), thus “3” will reach its maximum peak earlier as its peak is lower than “6” and a certain phase difference appears. For S1 > S2 (>S2′) > 0 = S3, this mode shifts from the supposed anti-phase with a certain difference due to the asymmetry in S2 and S2′. When the distance is far enough, coupling strength become negligible and results in incoherence of phase, which has a monotonous changing phase difference caused by the different inherent frequency for “3” and “6”, rather than the barely changing phase difference in symmetric system.

In a similar way, simulation curves and real profiles of the temperature distribution are plotted and show consistency with our model. Our model could apply to this case as well: closed enough oscillators more affected by radiation lead to in-phase mode; larger distance requires the system to keep an anti-phase alike mode to remain its stability; oscillators lose their coherence when distance is large enough.

**Figure 10:**
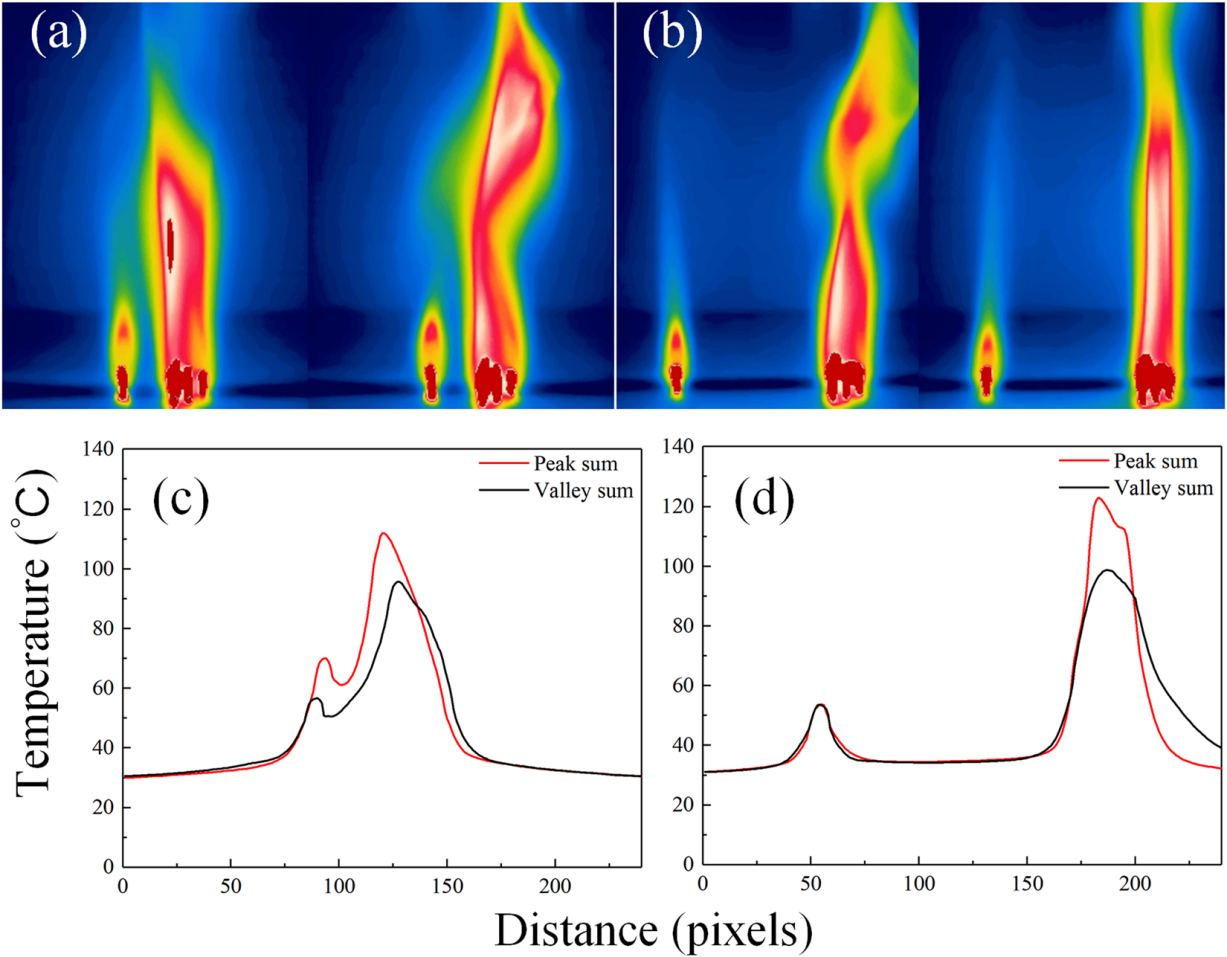
(**a**,**b**) The infrared images and (**c**,**d**) the temperature distribution in horizontal direction. (**c**) When the distance is near (20 mm), the single candle flame is affected by the radiation of “6” and starts to oscillate. The temperature in the middle space between two flames is distinctly higher than the ambient temperature. (**d**) When the distance is large (60 mm), the coupling strength is negligible and the single candle flame remains stable with no oscillation. The temperature between them is close to the ambient temperature.

In the end of this section, the “1 + 6” pattern is discussed, whose asymmetry is much more distinct than the case of “3 + 6”. As observed before, a single candle flame does not oscillate and keeps stable in an isolated situation. However, when an oscillator of “6” is placed nearby (<15 mm), the “1” starts to oscillate which is caused by the coupling from “6”, and exhibits synchronization close to the in-phase, similar to the case of “3 + 6”. As the distance gets larger, somewhere between 15 mm and 45 mm, the amplitude of “1” oscillation decreases to a small value and displays anti-phase synchronization. When the distance is larger than 45 mm, the coupling becomes so weak that the flame of a single candle stops oscillating and regains its stability. Meanwhile, the group of “6” oscillates still. The related time series are shown in Fig. 9(b–d) and the temperature distributions in Fig. 10. As the distance increases, the temperature in the middle between the two flames decays to the ambient temperature, indicating that the effective coupling through radiation becomes negligible.

### Discussion on the changes of phase difference in coupled systems

In Section 3.2 and 3.3, several changes of phase difference have been observed in differently coupled systems, which can be generally categorized into two cases: (1) the incoherent phase, which is caused by a rather weak coupling. (2) The discretely changing phase, which forms envelopes in time series and displays steps in phase difference. Their distinction and origin will be discussed in the following section.

The first case of phase changing is due to the long distance between the flames, which leads to a too weak coupling to keep coherence. For the ideal symmetric system, phase difference should remain constant even the distance between the oscillators is large, since the inherent frequency of the oscillators are the same. However, some tiny variation in phase difference is observed in our experiment, which changes slowly in half of a period (keeping within a range of *π*). Based on the observation and analysis, this kind of changes is attributed to the unstable combustion of the candle. As the flame lasts over 10 seconds, the wicks of the candles which participating in the combustion elongates and leans outwards, hence the flame loses its symmetry and tightness and gives rise to the irregularity in oscillation. The subtle change in amplitude will cause variations in frequency and phase difference as well. For the asymmetry system, it is clear that the phase difference should change monotonically since the inherent frequencies of non-identical oscillators are different as is observed in our experiments.

**Figure 11:**
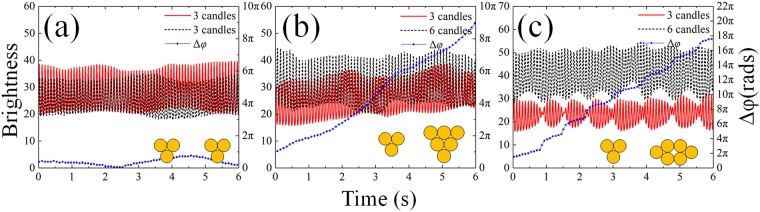
Comparison of multiple types of changing of phase difference. Red solid and black dashed lines for the time series of two oscillators and blue dotted-lines for the phase difference. (**a**) Symmetric system of “3 + 3” at a distance of 80 mm. The amplitude of each group fluctuates slightly and the phase difference changes subtly. (**b**) Asymmetric system of “3 + 6” at a distance of 55 mm. Though amplitudes change barely, the phase difference increases monotonically since the inherent frequencies are different. (**c**) Another arrangement of the asymmetric “3 + 6” system at a distance of 30 mm, which is illustrated by the yellow dots at lower right corner. In this case, amplitudes of both groups exhibit periodic envelopes and phase difference increases with “steps”.(see Supplementary Vid. S5)

In the second case, more interesting changes of phase difference are observed in our experiments. Another asymmetric system of “3 + 6” is considered, as shown in Fig. 11(c). Amplitudes of both oscillators exhibit periodic envelopes. The changing rate of phase in this case is much higher than in the first case, nearly twice as much. This kind of continuous change of phase difference is probably attributable to the periodic envelopes of the amplitude, which indicates a periodically changing frequency.

### Numerical modeling method

The computational fluid dynamic simulator Fire Dynamics Simulator (FDS), developed by NIST, was used to model the fire behaviors. The simulated results were compared and evaluated based on the visual illustration of the flame shape as well as the temperature distribution around the flame tip.

The heat-related parameters used in the simulation model are fixed at certain values and might not be totally in line with the actual situations due to the lack of heat flux measuring equipment. First we simulated the situation corresponding to section 3.2. In order to obtain the appropriate initial values for the simulation of a single group of candles, we used a method which is similar to the one in section 3.1, which the Heat Release Rate Per Unit Area (HRRPUA) of the burning part in the model was continuously adjusted to find out the minimum applicable parameters for the group. We also conducted simulations of other circumstances to observe the result.

For the simulation, a domain of 140 × 60 × 200 mm^3^ containing 210000 cells was created around the virtual candle. The boundary condition was set as opening vents for the 4 side walls and the ceiling of the candle and as cold inert wall for the floor. The model of candle was simplified to reduce the consumption of computing resources, which consists of an inert candle base of 11 × 11 × 20 mm^3^ and a wick of 5.5 × 5.5 × 10 mm^3^. The base and the wick are coaxially-aligned and the surfaces of the wick share a uniform HRRPUA of 1340.0 kW/mm^2^ by default. Also, the properties of the burning wax were taken from former measuring results. The initial parameters of the two candles are set as identical at the beginning of the simulations.

**Figure 12:**
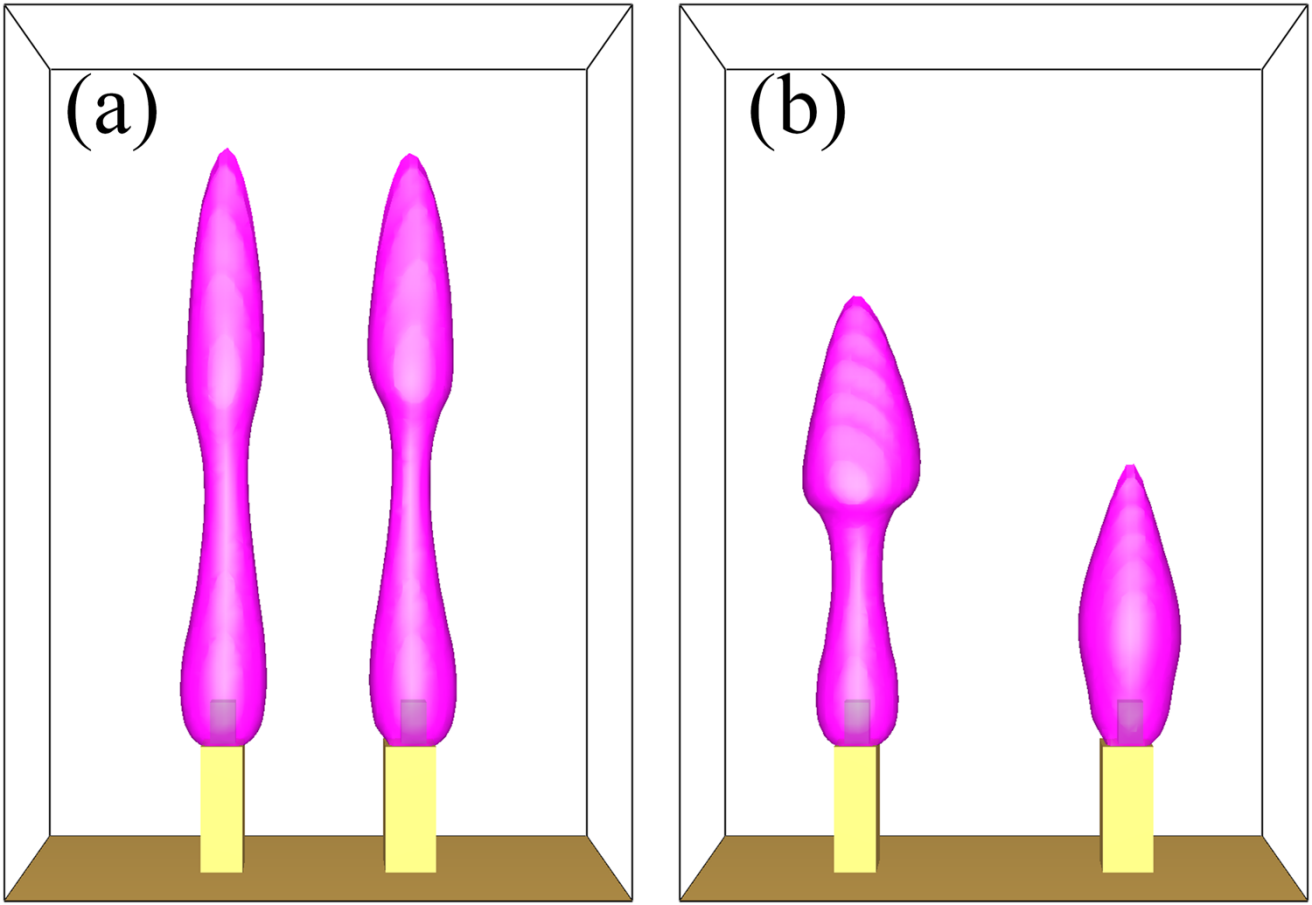
A snapshot of FDS simulation results for in-phase and anti-phase synchronizations. (**a**) In-phase mode at 30 mm and (**b**) anti-phase mode at 45 mm. Both figures share the parameters mentioned above and the flame regions are represented with 3D temperature contour surface (pink) approximately.

The same process for two identical oscillators was then repeated in the simulation. The results are shown in Fig. 12. As the distance between them increases, we found in-phase and anti-phase oscillations at 30 mm and 45 mm. Also when the distance is greater than 70 mm, the oscillators become incoherent, which is similar to the experimental results. The simulation verified that synchronization modes could change along with the increment of distance. The similarity between the results of experiments and simulations also stands as a verification for the proposed phenomenological model.

## Conclusion

In this report, single-group candle flame oscillators and coupled oscillators have been discussed both experimentally and theoretically. When three or more candles are closely bound into a group, the flame starts to oscillate. With the increasing number of candles, the oscillating frequency linearly decreases and the temperature increases. For a coupled system of two identical oscillators, whether it will lead to the in-phase, anti-phase and incoherent oscillation depends on the distances between the flames. Numerical simulation is consistent with experiments. For isosceles but non-equilateral triangle arrangements, experiments are carried out and explanations based on radiation are proposed for the variation of flame amplitude of the apex candle. For non-identically coupled groups, the same explanation applies, while the asymmetry induces phase shifts on the perfect in-phase or anti-phase synchronization. A phenomenological model based on the infrared temperature distribution, the “overlapped peaks model”, is proposed, which successfully explains all experimentally observed states in both symmetric and asymmetric cases, including a few interesting cases of phase difference evolution found in our experiments. The model reveals the relationship between synchronization states and the distances between oscillators, and successfully explained the underlying mechanism of synchronization state transition. Furthermore, the fire dynamics simulator (FDS)^26^ have been successfully used to model the behavior of candle flames and yielded valuable results in the simulation, which reasonably agree with the experimental results.

## Electronic supplementary material

S1 in-phase synchronization at 20 mm

S2 anti-phase synchronization at 35 mm

S3 Schlieren video of in-phase synchronization

S4 3+6 phaselock synchronization

S5 3+33 phaseshift synchronization

## Data Availability

The datasets generated during and/or analysed during the current study are available from the corresponding author on reasonable request.
